# Complications common in motorized intramedullary bone transport for non-infected segmental defects: a retrospective review of 15 patients

**DOI:** 10.1080/17453674.2021.1910777

**Published:** 2021-06-02

**Authors:** Mindaugas Mikužis, Ole Rahbek, Knud Christensen, Søren Kold

**Affiliations:** a Department of Orthopaedics, Aalborg University Hospital, Aalborg; b Interdisciplinary Orthopaedics, Aalborg University Hospital, Aalborg, Denmark

## Abstract

Background and purpose — Since the introduction of intramedullary bone transport nails only very few cases have been reported in the literature. Thus we evaluated the results and complications in a single institution retrospective cohort.

Patients and methods — 15 (median age 40 years (18-70), 8 males) consecutive patients, were included and the electronic patient records and radiographs were reviewed. Complications were severity graded and categorized as device or non-device related.

Results — The segmental bone loss was due to non-union site in 8 femurs and 4 tibias, or traumatic bone loss in 2 femurs and 1 tibia. The segmental bone defect was a median of 3 cm (0.5–10). 9 of 10 femoral cases and 4 of 5 tibial cases healed with the bone transport nail. All 15 patients had a healed docking site and regenerate at the end of treatment after a median of 13 months (6–38). 24 complications (15 device related and 9 non-device related) occurred in 11/15 patients with a minimum follow-up of 6 months after nail removal. The number of unplanned surgeries due to device related complications was: 0 in 9 patients, 1 in 3 patients, 2 in 1 patient, 3 in 2 patients.

Interpretation — Segmental bone defects can heal with a bone transport nail. However, the number of complications was high and 15 out of 24 complications were devicerelated. Optimizing nail design is therefore needed to reduce complications in intramedullary bone transport.

The concept of intramedullary bone transport nails to treat lower limb segmental bone defects was introduced by Baumgart et al. (1997) and refined by Kold and Christensen (2014) to alleviate the known complications seen in bone transport by external fixation (Paley and Maar 2000). The assumed advantages of using a fully implantable bone transport nail compared with external fixation is that early full joint motion is facilitated as skin and muscles are not transfixated, patient discomfort is reduced, pin site infections are eradicated, and the nail can be left in situ until the callus is sufficiently hardened. This potentially reduces the risk of fracture and secondary deformity as seen after removal of external fixators (Liu et al. 2020). However, only 5 cases of intramedullary bone transport nails have been reported (Baumgart et al. 1997, Kold and Christensen 2014, Accadbled et al. 2019), and a recent systematic review has showed high complication rates in bone lengthening despite the use of externally controlled motorized bone lengthening nails (Frost et al. 2020). Therefore, there is a need to investigate assumed advantages of the internal bone transport technique and observe if other complications are introduced by this new technique. We report our experience with the FITBONE bone transport nail in 15 patients with a minimum of 6 months’ follow-up after nail removal. We posed 2 questions: Are the bone transport nails capable of obtaining bone healing? Have new complications been introduced by the motorized transport nail?

## Patients and methods

### Design and participants

This is a single institution (Aalborg University Hospital, Denmark) retrospective case series with 15 patients (10 femur and 5 tibia) treated with the intramedullary bone transport FITBONE nail between 2012 and 2016. All bone transport nails were removed after the regenerate and docking site had fully consolidated in 3 out of 4 cortices. Follow-up after nail removal was median 46 months (6–89). Complications were extracted from patient records and scored according to Black et al. (2015) as categorized in Table 2. Complications were furthermore rated as device related or non-device related where device-related complications arise from properties of the implantable device itself (Lee et al. 2017).

The latest radiographs after nail removal were used for measurement of alignment (mechanical axis deviation, MAD) and limb length discrepancy (LLD). The long standing radiographs were obtained in 13 out of 15 patients. In the 2 other patients the LLD was evaluated clinically, and the alignment evaluated on regular radiographs.

The indication for bone transport with FITBONE nail was segmental bone loss, where it was judged safe to insert an intramedullary nail. Thus, the patients included in this study did not have soft tissue defects or preoperative clinical signs of infection. Bone biopsies were taken from the resection site for bacterial cultures during the nail insertion surgery.

The segmental bone loss was due to resection of non-union site in 8 out of 10 femoral cases and 4 out of 5 tibial cases, or traumatic segmental bone loss in 2 femoral cases and 1 tibial case. In the investigated time period, the femoral bone transport was only performed at our institution by the reported intramedullary FITBONE nail. In contrast, the tibial cases represent selected cases as the majority of tibial bone transports were made with external frames. In this patient case series, joint fusions were not performed. At least 2 surgeries had been performed prior to the bone transport in 12 out of 15 patients. A post-study description of the non-unions made from the Calori non-union score (Calori et al. 2008) showed a median of 35 (8–40). 13 out of 15 patients had a Calori non-union score above 25 indicating the need for bone transport (Abumunaser and Al-Sayyad 2011). 2 patients had a Calori score below 25 (patient no. 14: score of 8 and patient no. 15: score of 20).

### Treatment and surgery

**Figure 1.**
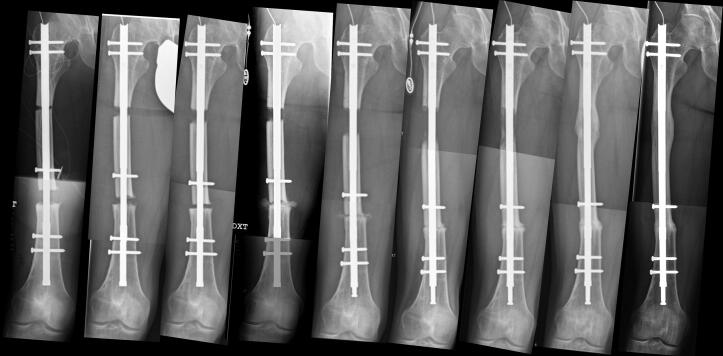
36-year-old male (patient 3), treated for atrophic non-union in the femur. After resection of non-union and proximal osteotomy, the transport nail is inserted (A), and the distraction and bone transport is started for the bone transport phase (A, B, C). When bone transport is completed and bone ends at the resection site are docked (D), compression at the docking site and additional bone lengthening is started for the bone lengthening phase (D, E, F). The protrusion of the distal tip of the nail shows the amount of additional lengthening of the femur (F). At the end of the consolidation phase (G, H), the regenerate and docking site are healed (I).

**Figure 2.**
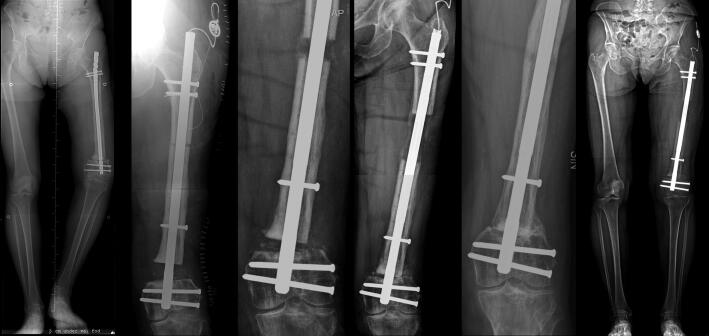
62-year-old woman (patient 5) with distal femur non-union, unsuccessfully treated with locking plate and IM nail prior to the bone transport surgery, with 6 cm LLD and severe varus deformity (A). The non-union was resected and deformity corrected at the same time. The distal segment length was 4 cm (B). Bone transport is almost completed and the gap is bone grafted (C). After docking is achieved, the distraction is continued and sliding mechanism of the proximal part of the nail provide lengthening of the femur (D). at 12 months’ follow-up, the docking site and regenerate are healed (E). Latest follow-up shows corrected mechanical axis and 1 cm LLD (F).

**Figure 3.**
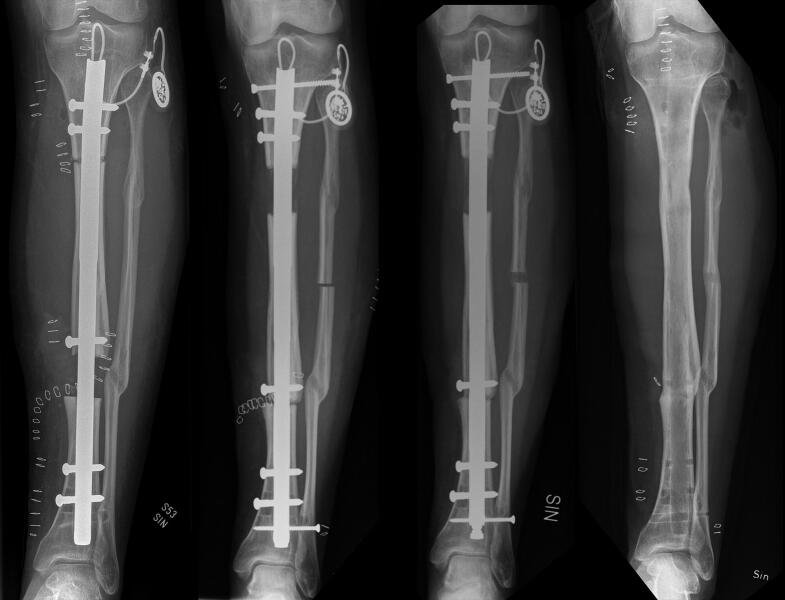
29-year-old female (patient 13) with 1 cm LLD and shortened fibula is treated with resection of nonunion (A). Proximal osteotomy for segmental bone transport was performed and 3 cm bone gap in the midshaft is closed, and the bone transport phase is completed (B). Prior to the lengthening of the tibia and fibula, a fibula osteotomy is performed (B), and proximal and distal tibio-fibular screws are inserted to protect the tibio-fibular joints. 1 cm lengthening of the tibia and the fibula is achieved (C). Follow-up after nail removal shows healed docking site and regenerate (D), LLD is corrected.

The intramedullary bone transport FITBONE nail (femur FSA, tibia TSA) was produced by Wittenstein intens GmbH (Igersheim, Germany). The FITBONE bone transport nail builds on the technology of the FDA-approved FITBONE lengthening nail. The FITBONE bone transport nail is CE-marked for the European market, but it is currently not FDA approved. The nail consists of a motorized lengthening device which has an up to 8 cm (depending on nail length) sliding slot in the middle part of the nail with a hole for locking screw (Figure 1A). The transporting bone segment is locked in the sliding hole between the osteotomy and resection site. Additional lengthening after completion of bone transport is obtained either by sliding locking screws (Figure 2) or protrusion of distal part of the nail as the total nail and bone segment length increase (Figure 1D–1F, Figure 3C). In tibial cases, the tibio-fibular joints were transfixated and a fibular osteotomy was perfomed prior to the lengthening phase of the tibia and fibula. The 8 cm maximum stroke of the nail can be distributed between the transport of the segment and bone segment lengthening.

Surgery consisted of 3 steps: (1) resection of non-union or non-vital bone ends; (2) ventilating drill holes, bone canal reaming, and a percutaneous osteotomy for distraction osteogenesis; (3) insertion of nail and locking in both ends, and insertion of the sliding screw in the bone transport segment (Figure 1). Bone malalignment in any plane was corrected acutely at the time of nail insertion. The reverse planning method (Baumgart 2009) was used to maintain or to correct bone malalignment in the frontal plane (Figure 2). On the femur, the use of an antegrade or a retrograde approach depends on the location of the defect, presence of deformity and the nail design. The segmental bone defect was median 3 cm (0.5–10).

13 cases were grafted with either autogenous bone graft, Osigraft (BMP-7) or both (Table 1). In the cases where grafting was postponed until docking of the transported segment, a percutaneous docking procedure was performed with removal of fibrous tissue prior to grafting.

**Table 1.** Summary of the 15 patients treated with FITBONE bone transport nails

Case no.	Age	Original pathology	Nail approach	Type of graft	Union at docking site	Healing of bone regenerate	Follow-up in months after nail removal
Femur							
1	61	Non-union, oligotrophic	Retrograde	Autograft + Osigraft	No	Yes	70
2	56	Non-union, oligotrophic	Retrograde	Autograft + Osigraft	Yes	Yes	47
3	36	Non-union, oligotrophic	Antegrade	Autograft + Osigraft	Yes	Yes	59
4	23	Non-union, atrophic	Retrograde	Autograft	Yes	Yes	61
5	62	Non-union, atrophic	Antegrade	Autograft + Osigraft	Yes	Yes	42
6	70	Non-union, atrophic	Antegrade	Autograft + Osigraft	Yes	Yes	35
7	22	Segmental bone loss, open fracture	Antegrade	No graft	Yes	Yes	28
8	37	Non-union, atrophic	Retrograde	Autograft + Osigraft	Yes	Yes	26
9	18	Segmental bone loss, open fracture	Antegrade	No graft	Yes	Yes	46
10	62	Non-union, hypertrophic	Antegrade	Autograft + Osigraft	Yes	Yes	29
Tibia							
11	53	Non-union, oligotrophic	Antegrade	Osigraft	Yes	Yes	89
12	40	Segmental bone loss, open fracture	Antegrade	Autograft	No	Yes	58
13	29	Non-union, atrophic	Antegrade	Osigraft	Yes	Yes	46
14	38	Non-union, hypertrophic	Antegrade	Autograft + Osigraft	Yes	Yes	6
15	40	Non-union, hypertrophic	Antegrade	Autograft	Yes	Yes	27

### Aftercare

The physiotherapy was started at day 1 after surgery with up to 20 kg weight-bearing during the distraction phase, and thereafter full weight-bearing was allowed. Bone distraction started after median 10 days (5–12) following surgery. The distraction speed was initially 0.33 mm 3 times per day, which was adjusted during the lengthening period depending on the quality of the bone regenerate. During the distraction phase the bone regenerate was radiographically followed every 1 or 2 weeks. After the end of distraction, bone healing of the regenerate and docking site was monitored monthly until full consolidation.

### Ethics, funding, and potential conflicts of interest

The study was approved by the institutional review board, registration ID number 2020-157.

Each author certifies that he or she has no commercial associations or received funding that might pose a conflict of interest in connection with the submitted article.

## Results

### Demographics

8 males and 7 females were included with the median age being 40 years (18–70). 4 patients had comorbidities such as diabetes, severe obesity, lupus, and rheumatoid arthritis. Both smokers (5 patients) and non-smokers (10 patients) were included. Smokers were recommended to quit smoking prior to surgery, but no control was performed.

Preoperative LLD was median 2 cm (0–6). Preoperative MAD was from 88 mm varus to 7 mm valgus (median 19 varus).

In the femur group, 1 patient (patient 5) had a preoperative knee range of motion (ROM) from 0° to 40° and was subsequently treated with Judet’s quadricepsplasty (Ali et al. 2003) at the end of bone transport and lengthening.

### Bone healing (Table 1)

**Figure 4.**
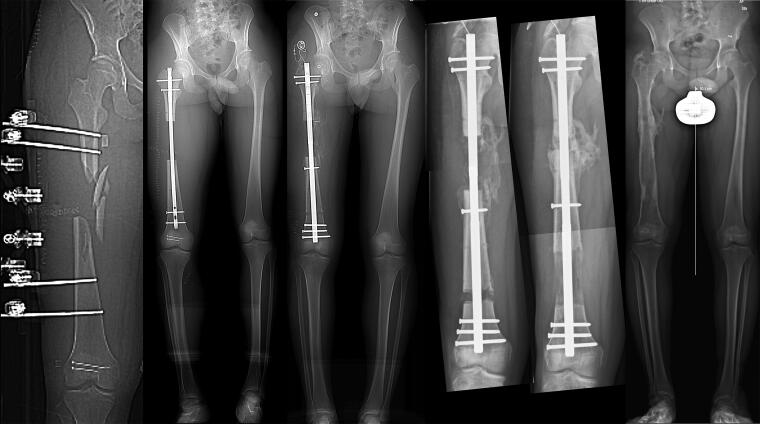
Failure of femoral docking in a 61-year-old woman (patient 1). The position of the locking screw in the transport segment is 7 mm distally to the osteotomy site (A). The bone transport is almost completed, the compression of docking site and additional lengthening is started (B). The compression at the docking site failed (C) as the transport screw cut out and the transport segment lost the distraction.

9 out of 10 femoral cases healed with the bone transport nail. The femoral failure occurred in a 61-year-old woman with impaired bone quality due to gastric bypass (patient 1, Table 1). The transport screw was inserted too close to the resection site, leaving only 7 mm of pulling bone stock proximal to the screw. As compression was applied over the docking site, the screw cut through the 7 mm bone stock resulting in loss of the achieved bone transport and thereby loss of bone contact at the docking site (Figure 4). The nail was changed to a regular trauma nail, and the femur healed with 3 cm shortening. LLD was later corrected by a standard FITBONE lengthening nail. 4 out of 5 tibial cases healed with the bone transport nail. The tibial case that did not heal was initially treated for acute bone loss after an open Gustilo IIIA fracture and developed signs of infection at the bone defect site during bone transport (patient 12, Table 1). Therefore, the nail was converted to an external circular frame after completion of the bone transport, and uneventful healing occurred hereafter. At the latest follow-up with median of 46 months (6–89) after nail removal all 15 patients had healed bone docking site and regenerate.

**Figure 5.**
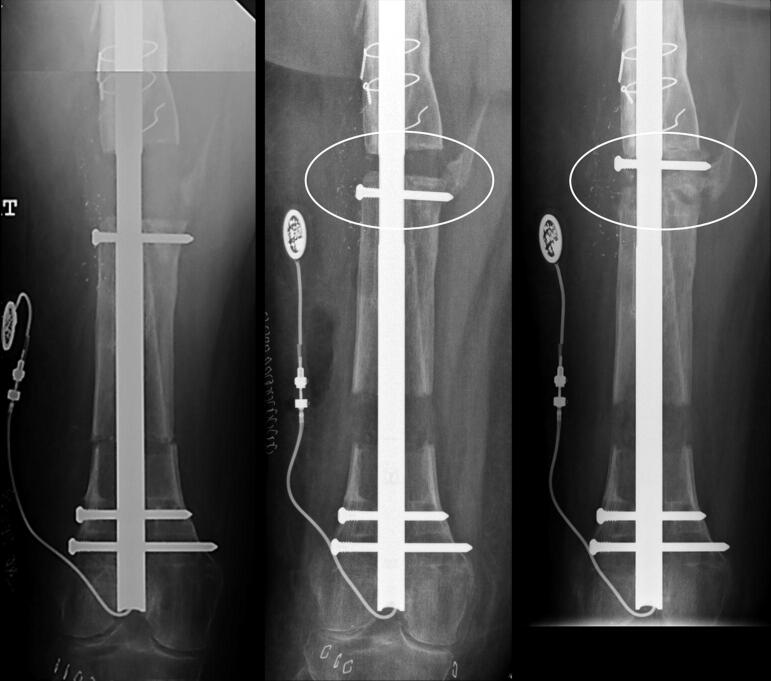
22-year-old patient (patient 7) with open fracture and segmental bone defect, primarily treated with external fixation (A). After resection of non-vital bone, the femur with 10 cm bone defect was stabilized with an IM nail (B). The trauma nail is changed to a bone transport nail and distraction is started (C). Bone transport at 2 cm (D), and completed at 8 cm due to early callus formation at the docking site (E). Follow-up after nail removal (F), the remaining varus of proximal tibia is not changed.

2 femoral cases did not receive any docking procedure as radiographic signs of good callus formation were present at the time of docking (Figure 5E). In the remaining cases, the docking site was grafted (Table 1).

### Complications (Table 2)

24 complications occurred in 11 out of 15 patients with a minimum follow-up of 6 months after nail removal. 2 complications led to minimal change in treatment plan (category I) in 2 patients. 16 complications led to substantial change in treatment plan (category II) in 9 patients. 3 complications resulted in failure to achieve treatment plan (category IIIA) in 3 patients. 1 complication (fracture after nail removal) resulted in new pathology (category IIIB) in 1 patient. 2 complications (reduced knee flexion and neurogenic foot pain) resulted in permanent sequelae at the end of treatment (category IIIB) in 2 patients. 19 unplanned surgeries (11 device related and 8 non-device related) were needed in 10 out of 15 patients. Infections of the receiver occurred 5 times in 3 patients, rated as category II device-related complications. Infected receivers were changed and antibiotics administered based on biopsy for cultures. In 1 patient synovectomy was performed as the infection had spread via the connection cable from the receiver to a retrograde-inserted femoral nail into the knee joint.

**Table 2.** Complications graded by severity (I to IIIB) according to Black et al. 2015 and by origin (device and non-device related) according to Lee et al. 2017

Case no.	Device related complications, categories a				Non-device related complications, categories a				Unplanned surgeries, n
	I	II	IIIA	IIIB	I	II	IIIA	IIIB	
Femur									
1	2 receiver change due to infection, 1 screw discomfort						1 loss of transport, changed to trauma nail		4
2	1 receiver removal due to discomfort								1
3									0
4									0
5	1 nail re- bounding	2 receiver change due to infection 1 screw removal due to discomfort						1 fracture after removal	3
6	1 screw backing out (changed during docking surgery)								1
7									0
8	1 screw backing out								1
9								1 stiff knee	0
10	1 receiver change due to infection	1 nail stopped to lengthen	1 additional grafting						3
Tibia									
11	1 screw backing out (changed during docking surgery)		1 syndesmotic screw removal due to discomfort						1
12	1 screw backing out		1 infection debridement	1 changed to external fixation					3
13									0
14	1 nail re- bounding							1 neurogenic pain, tarsal tunnel release	1
15			1 insertion of forgotten syndes- motic screw						1
Complications in total (n = 24)									
2, distraction 8, other 4, stability			1, distraction	0	0	4	2	3	
Unplanned surgeries in total									19

a Category I complications required minimal intervention, and treatment goal was still achieved. Category II needed substantial change in treatment plan, such as unplanned return to operating room; the treatment goal was still achieved. Category IIIA complications failed to achieve treatment goal, but without developing new pathology or permanent sequelae. Category IIIB complication failed to achieve treatment goal and/or new pathology or permanent sequelae developed.

### Other relevant findings

The total distraction of the nail was median 4 cm (2–8), including the bone transport and additional lengthening (Table 3). Final LLD was 0 mm in 4 patients, < 10 mm in 10 patients and 3 cm in 1 patient after bone transport nail removal. The patient with 3 cm LLD was a femoral case (patient 1, Table 3) with transport screw cut-out and after healing with a regular trauma nail. The LLD is reported prior to additional lengthening with a standard FITBONE lengthening nail.

**Table 3.** Data summary of 15 bone transport patients

Case no.	Preoperative MAD, mm	PreoperativeLLD, cm	Resection size, cm	Bone transport	Additional lengthening, cm	Final LLD, cm	Postoperative MAD, mm	Knee ROM
Femur								
1	–7	1	3.5	2.5	–2	3	0	0–100
2	21	2.5	2.5	2.5	1.5	1	16	0–135
3	0	3	2.5	2.5	2.5	0.5	0	0–140
4	12	0.5	3	3	0	0.5	0	0–140
5	88	7	2	2.5	3.5	1	13	0–80
6	33	3	2	2.5	1	1.5	24	0–130
7	17	0.5	10	8	0	1.5	29 (deformity at tibia)	0–120
8	27	0.5	4.5	5	0.5	1	27	0–120
9	0	4	4	1.5	4	0	0	0–85
10	30	2.5	4	4	0.5	2	–5	0–110
Tibia								
11	23	2	3	3	2	0	9	0–140
12	MPTA 89°	0	8	8	0	2	MPTA 87°	0–140
13	10	1	3	3	1	0	10	0–140
14	43	1	2	2	1	0	MPTA 81°	0–140
15	–4	1.5	0.5	0.5	1.5	0	7	0–140
Median (range)		2 (0–7)	3 (0–10)	3.5 (1.5–8)	1 (–2 to 3.5)	1 (0–3)		

LLD = limb length discrepancy

MAD = mechanical axis deviation

MPTA = medial angle between the tibial mechanical axis and the proximal articular surface of the tibia in the coronal plane

The bony deformity at the end of treatment was within 5 degrees in any plane (coronal, sagittal, and axial) in 14 out of 15 patients. 1 patient (patient 5, Table 3) had a sagittal plane deformity of 10°.

## Discussion

### Background and rationale

To our knowledge, this study represents the largest case-series (Accadbled et al. 2019, Baumgart et al. 1997, Kold and Christensen 2014) of patients treated with a bone transport nail. The 15 patients had segmental bone defects due to either acute traumatic bone loss or bone resection of non-united fractures. Bone healing was achieved with the FITBONE bone transport nail in 9 out of 10 femoral cases and in 4 out of 5 tibial cases. In comparison, Accadbled et al. (2019) reported successful healing with the FITBONE bone transport nail in 3 out of 3 femoral segmental bone defects after tumor resection. No tibial case series have been presented for bone transport nails, but in a recent systematic review of Ilizarov bone transport for treatment of tibial defects, the mean bone union rate was 90% (77–100) (Aktuglu et al. 2019).

In a retrospective analysis of complications in 282 consecutive cases treated with Ilizarov external bone transport in the lower extremity, pin tract infections occurred in 66% of patients (Liu et al. 2020). In the majority, the pin tract infections were managed by daily pin site care and oral antibiotics; however, 20% of patients suffered deep pin tract infection or pin loosening and underwent treatment by pin replacement and intravenous antibiotics. Such pin-tract infections are avoided in our study by the use of a fully implantable bone transport nail. However, the complication rates were still high with this new treament, when patients were followed up to a minimum of 6 months after nail removal. 24 complications were observed in 11 out of 15 patients and 19 unplanned surgeries were performed in 10 out of 15 patients. Only 4 out of 15 patients did not sustain any complications, and 10 out of 15 patients had to undergo unplanned surgeries for complications. In 2 out of 15 patients the complication resulted in permanent sequelae. Lee et al. (2017) argued that, to fully understand the pros and cons of new bone-lengthening devices, analyses should divide complications related to the device itself from those that are not associated with the device. 15/24 complications were device related. Because of the novelty of the FITBONE transport nail this is expected and we believe that further development of bone transport nails could reduce these complications. As an example, 5 out of all 24 complications were related to infection of the subcutaneous receiver. This rate of infection seems high in light of our experience with FITBONE lengthening nails and a recent systematic review of complications using lengthening nails (Frost et al. 2020). The high number of infections associated with the receiver in our study might be a result of recurrence of infection at the receiver site after exchange of the receiver in 2 patients. However, these infections will not appear if bone transport nails without such a receiver are used. Furthermore, 6/24 of complications that arose from backing of locking screws might be reduced by optimising screw design. However, the most severe complications, resulting in 2 permanent sequelae and 1 new pathology, were non-device related.

Our current approach for treating segmental defects differs between the femur and the tibia. All femoral cases are treated by nails to avoid final treatment with external fixators. In cases of clinical infection or compromised soft tissues, extensive debridement is followed by temporal external fixation converted to an intramedullary nail within 2 weeks. Acute shortening is well tolerated on the femur, and segmental defects up to 4–6 cm are treated with a staged protocol. Acute shortening, autologous bone grafting, and standard intramedullary nailing allow for crucial early functional rehabilitation. When union has been obtained, the LLD is corrected at a second stage by standard intramedullary lengthening nail. Larger femoral defects of more than 4–6 cm are treated by femoral bone transport nails.

Segmental defects of the tibia in the presence of clinical infection or compromised soft tissues are treated with external bone transport in a circular frame. At our institution, composite bone and soft-tissue loss of the leg are treated without free flaps (El-Rosasy and Ayoub 2020), and the indications for tibial bone transport nails might differ if immediate free flap coverage is provided of soft-tissue defects. We use a tibial transport nail for segmental defects in cases of uncompromised soft tissues where stable fixation can be obtained with the nail. When presence of infection is suspected, work-up with C-reactive protein level and PET-CT are performed. However, Moghaddam et al. (2015) found that 17% of non-unions, judged as aseptic, had positive intraoperative cultures. Therefore, we recommend thorough debridement when inserting bone transport nails, and in the case of unexpected positive cultures from resected bone, prolonged antibiotic treatment should be given (Kold and Christensen 2014).

Non-union patients tend to suffer significant LLD and in our cases 7 out of 15 patients had preoperative LLD of more than 2 cm. One of the advantages with the FITBONE transport nail is the capability of additional lengthening when the bone transport phase is finished. The leg length might then be equalized within 1 surgery and the same nail unit.

It is recommended by the company that the FITBONE transport nail is removed at the end of treatment, and it is mandatory that case-series should report on complications after recommended nail removal. We had a minimum of 6 months’ follow-up after nail removal. In a 70-year-old female a fracture occurred through the femoral regenerate 3 days after nail removal. This complication might have been avoided by exchanging the bone transport nail for a regular trauma nail, and this exchange technique was later performed in 3 patients. We remove all transport nails when the regenerate and the docking site have healed, and based on clinical judgement of refracture risk the need for exchange nailing is individualized. However, if explantation of nails was not needed, the need for secondary surgery and the risk of recurrent deformity and fracture might be lowered.

### Limitations

The retrospective design of our study might lead to inaccurate reporting of complications. Furthermore, the tibial cases represent highly selected cases as most tibial bone transport cases treated at our institution in the same time period were performed with an external circular frame. In contrast, we have performed femoral bone transport with a nail only since the introduction of the femoral FITBONE transport nail at our department in 2012, as it is known that complication rates for external bone transport are higher for femoral than tibial transport (Liu et al. 2020). The lack of patient-reported outcome measures makes it impossible to conclude to what extent the bone healing and additional lengthening did improve patient quality of life. Therefore, prospective studies with stringent treatment algorithms and registration of patient-reported outcome measurements are needed.

In conclusion, this retrospective case-series showed that segmental bone defects healed with a FITBONE bone transport nail in 13 of 15 cases. By introducing the motorized nail the number of device-related complications was high. Future research should focus on reducing device-related complications by optimizing nail design.

Conceptualization: MM, OR, SK. Data extraction: MM, SK. Surgery: MM, KC, SK. Supervision: OR, SK. Writing original draft: MM. Writing, reviewing, and editing: MM, OR, KC, SK.

*Acta* thanks Jan Duedal Rölfing and other anonymous reviewers for help with peer review of this study.
